# YB-1 Interferes with TNFα–TNFR Binding and Modulates Progranulin-Mediated Inhibition of TNFα Signaling

**DOI:** 10.3390/ijms21197076

**Published:** 2020-09-25

**Authors:** Christopher L. Hessman, Josephine Hildebrandt, Aneri Shah, Sabine Brandt, Antonia Bock, Björn C. Frye, Ute Raffetseder, Robert Geffers, Monika C. Brunner-Weinzierl, Berend Isermann, Peter R. Mertens, Jonathan A. Lindquist

**Affiliations:** 1 Clinic of Nephrology and Hypertension, Diabetes and Endocrinology, Otto-von-Guericke University, 39120 Magdeburg, Germany; c.hessman@gmx.net (C.L.H.); josephine.hildebrandt@freenet.de (J.H.); aneri.shah@ovgu.de (A.S.); Sabine.Brandt@med.ovgu.de (S.B.); antonia.bock94@t-online.de (A.B.); 2 Department of Nephrology and Clinical Immunology, RWTH Aachen University, 52074 Aachen, Germany; bjoern.christian.frye@uniklinik-freiburg.de (B.C.F.); uraffetseder@ukaachen.de (U.R.); 3 Genome Analytics Research Group, Helmholtz Centre for Infection Research, 38124 Braunschweig, Germany; robert.geffers@helmholtz-hzi.de; 4 Department of Experimental Pediatrics, Otto-von-Guericke University, 39120 Magdeburg, Germany; monika.brunner-weinzierl@med.ovgu.de; 5 Institute of Laboratory Medicine, Clinical Chemistry and Molecular Diagnostics, University Hospital Leipzig, 04103 Leipzig, Germany; berend.isermann@medizin.uni-leipzig.de

**Keywords:** Y-box binding protein-1 (YB-1), DNA binding protein-B (DbpB), progranulin, tumor necrosis factor alpha (TNFα), inflammation, immune modulation, signaling, macrophage

## Abstract

Inflammation and an influx of macrophages are common elements in many diseases. Among pro-inflammatory cytokines, tumor necrosis factor α (TNFα) plays a central role by amplifying the cytokine network. Progranulin (PGRN) is a growth factor that binds to TNF receptors and interferes with TNFα-mediated signaling. Extracellular PGRN is processed into granulins by proteases released from immune cells. PGRN exerts anti-inflammatory effects, whereas granulins are pro-inflammatory. The factors coordinating these ambivalent functions remain unclear. In our study, we identify Y-box binding protein-1 (YB-1) as a candidate for this immune-modulating activity. Using a yeast-2-hybrid assay with YB-1 protein as bait, clones encoding for progranulin were selected using stringent criteria for strong interaction. We demonstrate that at physiological concentrations, YB-1 interferes with the binding of TNFα to its receptors in a dose-dependent manner using a flow cytometry-based binding assay. We show that YB-1 in combination with progranulin interferes with TNFα-mediated signaling, supporting the functionality with an NF-κB luciferase reporter assay. Together, we show that YB-1 displays immunomodulating functions by affecting the binding of TNFα to its receptors and influencing TNFα-mediated signaling via its interaction with progranulin.

## 1. Introduction

Y-box binding protein-1 (YB-1) is a multifunctional protein, which was initially identified by its binding to Y-box motifs (inverted CCAAT box) within the promoters of human MHC class II genes [1]. YB-1 performs a wide variety of cellular functions and is involved in diverse biological processes, such as the regulation of transcription/translation, modification of chromatin, DNA repair, RNA packaging, and modulating cellular stress responses [2]. YB-1 controls cell cycle-dependent genes, and increased nuclear expression has been found in tumors [3,4,5]. In addition to its intracellular functions, YB-1 is secreted via a non-classical pathway following cytokine stimulation with either transforming growth factor β (TGF-β) or platelet-derived growth factor (PDGF) [6,7]. Extracellular YB-1 is found in the serum and urine of patients suffering from inflammatory glomerular disorders, as well as in the serum and urine of animals undergoing experimental models of inflammatory disease. Secreted YB-1 was shown to be chemoattractive and participates in monocyte/macrophage recruitment, differentiation, and function after lipopolysaccharide (LPS) stimulation *in vitro* and in an animal model of kidney inflammation *in vivo*, supporting its role in immunomodulation [8,9]. Recently, secreted YB-1 was identified as an important factor in melanoma [10]. In a previous study, we identified and characterized an interaction of extracellular YB-1 with receptor Notch-3, leading to intracellular signaling [7]. Notch-3 was shown to be upregulated in human kidney diseases and plays a role in modulating inflammation and fibrosis in tubulointerstitial kidney injury [11]. Extracellular YB-1 regulates Notch-3 receptor expression and signaling [12].

YB-1 is comprised of three domains with distinct functions [2]. The N-terminal domain is alanine/proline-rich and associates with actin. The cold shock domain features DNA/RNA binding activities that enable its transcriptional and translational activities. The C-terminal tail associates with other YB-1 proteins (homo-multimerization) and controls the subcellular distribution of YB-1 within the cell [13,14,15,16].

Tumor necrosis factor α (TNFα) promotes inflammation and tissue-destruction in a number of inflammatory diseases, including glomerulopathies [17,18,19,20,21,22,23,24]. TNFα acts through two distinct receptors. TNF receptor 1 (TNFR1) is widely expressed. Signaling via this receptor promotes pro-inflammatory responses and induces cell death, but it can also lead to cell survival [25]. In the healthy kidney, TNFR1 is mainly expressed on glomeruli and peritubular endothelial cells. TNF receptor 2 (TNFR2) expression is more restricted. It is primarily expressed on immune cells. In various kidney diseases, TNFR2 expression is induced on renal cells [26,27,28]. Signaling via TNFR2 is not completely understood, but it appears to have an immunomodulatory function [29,30].

Based on our current understanding of inflammatory diseases, new treatment strategies and drugs have been developed that target key cytokines or interfere with their signaling cascades. Common approaches include cytokine blocking monoclonal antibodies (Anakinra: IL-1β, Infliximab: TNFα) and soluble TNF receptors (Etanercept, Onercept) [31,32]. Treatment of rheumatoid arthritis patients with TNFα-blocking drugs improved clinical symptoms. Patients who also suffered from chronic kidney disease demonstrated a beneficial effect on kidney function [24]. Although all anti-TNFα therapies have well-demonstrated efficacy, the increased risk of lymphomas or reactivation of latent infection remains a drawback [32,33,34,35,36]. Therefore, drug development has more recently focused on interference with cytokine receptors, thereby blocking the activation of cytokine-induced signaling pathways.

## 2. Results

### 2.1. YB-1/PGRN Interaction

**Figure 1:**
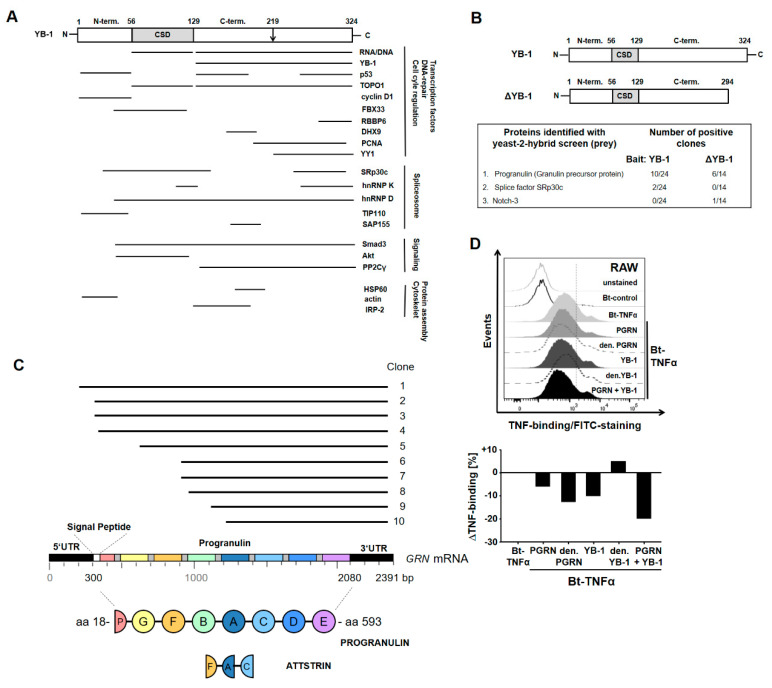
Identification of Y-box binding protein-1 interacting proteins. (**A**) Published interaction partners of Y-box binding protein-1 (YB-1) and the corresponding protein sequences that are needed for interaction. Figure modified from Eliseeva et al. 2011 [2]. (**B**) A cDNA library of human mesangial cells was screened with a yeast-2-hybrid screen for interaction partners of YB-1. A full-length YB-1 or a C-terminally truncated construct ΔYB-1 were used as bait. Identified interactions of YB-1 with receptor Notch-3 and SRp30c have been already published [7,37]. (**C**) Clones generated in the yeast-2-hybrid screen that were positive for YB-1 and progranulin (PGRN) interaction were picked and sequenced, allowing us to identify the binding sequence of the protein. (**D**) RAW 264.7 macrophages were pre-incubated with recombinant YB-1 and/or recombinant PGRN, stained for binding of Bt-TNFα (biotinylated tumor necrosis factor α) and analyzed by flow cytometry.

Intracellular YB-1 has a number of known interacting partners (Figure 1A) [2]. However, less is known about extracellular YB-1. In a previous effort to identify novel YB-1 interacting proteins, we performed a yeast-two hybrid (Y2H) screen using a cDNA library generated from a human mesangial cell line. Both full-length YB-1 and a C-terminally truncated ΔYB-1, missing 30 amino acids that includes the dilysine motif required for secretion (K301/K304), were used as bait (Figure 1B) [6]. The functional relevance of this approach has been demonstrated for two other interacting proteins: receptor Notch-3 and splicing factor SRp30c [7,37]. Among the proteins identified (see Figure 1C), progranulin (PGRN) was of particular interest, since it is thought to exert its anti-inflammatory activities by modulating the binding of TNFα to its receptors [38,39]. Therefore, we hypothesized that YB-1 and PGRN might team up to modulate TNFα activity.

The finding that YB-1 directly binds to PGRN prompted us to investigate whether YB-1/PGRN show an added benefit to modulating the TNFα–TNFR interaction. Therefore, we performed a flow cytometric binding assay and tested the effects of adding either recombinant PGRN and/or recombinant human YB-1 (rhFlag-YB-1) on the TNFα–TNFR interaction. The incubation of RAW macrophages with rPGRN resulted in a decreased binding of biotinylated TNFα (Bt-TNFα) to TNFR, confirming that PGRN competes with TNFα for binding to its receptors (Figure 1D). Similarly, rhFlag-YB-1 also competes with TNFα for receptor binding. Together, PGRN and YB-1 show an enhanced inhibitory effect on TNFα binding. Heat denaturing YB-1 abrogated the inhibitory effect, thus demonstrating the specificity of this interaction. Surprisingly, heat-denatured PGRN showed enhanced inhibition, suggesting that a change in conformation might improve its ability to inhibit TNF binding.

### 2.2. YB-1 Inhibits TNFα-Binding to Its Receptors

**Figure 2:**
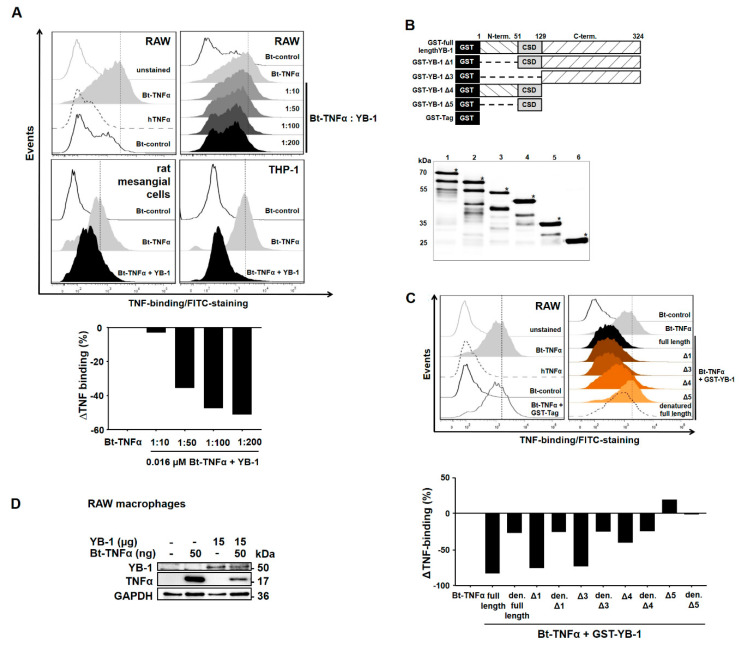
YB-1 inhibits TNFα binding to TNF receptors (TNFRs). (**A**) RAW 264.7 macrophages were pre-incubated with increasing doses of recombinant Flag-YB-1 and stained for Bt-TNFα-binding. Binding of Bt-TNFα was analyzed with flow cytometry. (**B**) GST-tagged YB-1 deletion mutants were used to identify the minimal sequence required for interaction. Western Blot analysis of purified GST constructs: lane 1—GST-full length YB-1, lane 2—GST-YB-1 Δ1, lane 3—GST-YB-1 Δ3, lane 4—GST-YB-1 Δ4, lane 5—GST-YB-1 Δ5 and lane 6—GST-tag alone. (**C**) RAW 264.7 cells were pre-incubated with GST-YB-1 deletion mutants, stained for the binding of Bt-TNFα, and analyzed with flow cytometry. (**D**) RAW 264.7 macrophages were incubated with 15 µg recombinant Flag-YB-1 and/or 50 ng/mL Bt-TNFα, washed, lysed and bound ligand detected by Western blotting. GAPDH is included as the loading control.

To confirm the observation that YB-1 alone competes with TNFα for receptor binding, we repeated the flow cytometric TNFα binding assay. We tested the effects of adding different doses of recombinant human YB-1 on the TNFα–TNFR interaction. The incubation of RAW macrophages with biotinylated TNFα (Bt-TNFα) confirmed that TNFα shows a specific binding to its receptors. The addition of unlabeled TNFα could compete away binding of the biotinylated ligand, while the addition of an unrelated biotinylated protein showed negligible binding (Figure 2A, upper left panel). As demonstrated in Figure 1D, rhFlag-YB-1 also competed with TNFα for receptor binding. The inhibitory effect of YB-1 turned out to be dose-dependent, as shown in Figure 2A (upper right panel). The inhibitory effect was not cell-specific, since a similar decrease in TNFα binding was seen using both human THP-1 cells and rat mesangial cells (Figure 2A, lower panels).

To determine the region(s) within YB-1 responsible for binding to the TNFR, we made use of previously published GST-tagged YB-1 deletion mutants to identify the minimal sequence required for the YB-1–TNFR interaction (Figure 2B) [40]. Flow cytometric analysis (Figure 2C) revealed that deletion of the N-terminal domain YB-1Δ1 had no negative affect on the ability of YB-1 to inhibit TNFα binding. Similarly, deleting the N-terminal and cold shock domain (CSD), i.e., GST-YB-1Δ3, shows a similar ability to inhibit TNFα binding, indicating that the C-terminal domain is sufficient for achieving a complete competition of TNFα-binding to the TNFRs. The deletion mutant GST-YB-1Δ4 containing both the N-terminal and cold shock domains shows a reduced ability to inhibit TNFα compared to the full-length construct, whereas GST-YB-1Δ5, containing only the cold shock domain, has lost the ability to inhibit TNFα binding. Denaturing YB-1 abrogated its inhibitory effect, thus demonstrating the specificity of this interaction. Thus, it appears that largely the C-terminal domain of YB-1 contributes to TNFR binding; nevertheless, a minor effect is also seen with the protein N-terminus. The cold shock domain itself does not appear to contribute to receptor interactions.

To visualize this effect, we incubated RAW 264.7 macrophages with biotinylated human TNFα (Bt-TNFα), which served as a positive control for the binding assay, and/or recombinant human YB-1. Figure 2D demonstrates that macrophages are able to bind TNFα or YB-1, indicating that these proteins bind to specific receptors on the cell surface. When both proteins are added together, the amount of TNFα recovered is strongly reduced, confirming that YB-1 competes with TNFα for binding to its receptor.

### 2.3. YB-1/PGRN Inhibits TNFα-Mediated Signaling

**Figure 3:**
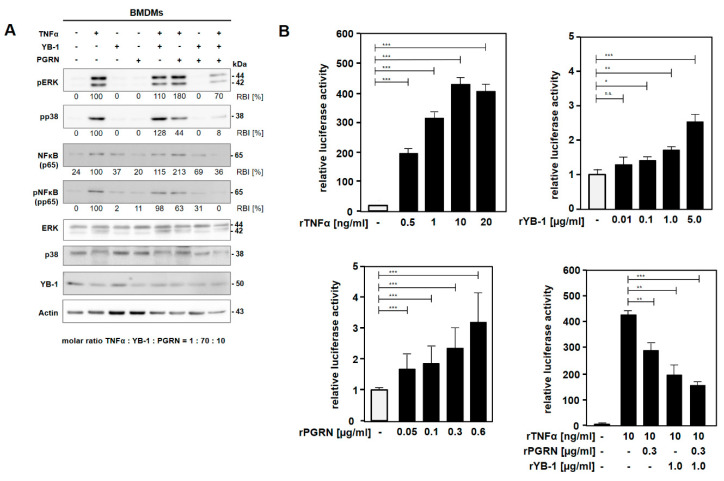
TNFα-mediated signaling is influenced by YB-1 and PGRN. (**A**) Bone marrow-derived macrophages (BMDMs) were stimulated with recombinant mTNF (625 pM), PGRN (5 nM), and YB-1 (28 nM) for 10 min. After cell lysis, proteins were blotted and analyzed for the expression of pERK, pp38, NF-κB, pNF-κB, YB-1, and actin as the loading control. Band intensities were quantified and normalized to actin. Values are expressed as relative band intensity (RBI), with the level of activation for TNFα-stimulation set to 100%. (**B**) The pGL4.32 luciferase reporter construct harboring an NF-κB responsive element was introduced into HEKLentiX cells by calcium phosphate precipitation. Recombinant hTNFα was added at the indicated concentrations following the transfer of cells into serum-free starving medium. Stimulation with recombinant proteins was performed as indicated for 5 h, and luciferase activity quantified according the manufacturer’s instructions. * *p* = 0.05, ** *p* = 0.01, *** *p* = 0.001.

To investigate whether YB-1/PGRN influences TNFR signaling, bone marrow-derived macrophages (BMDMs) were stimulated with murine TNFα, PGRN, and YB-1 alone or in combination. Cell signaling was analyzed for the expression and activation of extracellular signal-regulated kinase (ERK), p38, and nuclear factor kappa-B (NF-κB) (Figure 3A). Stimulation of wild-type BMDMs with TNFα induced a rapid phosphorylation of ERK, p38, and NF-κB p65. A similar level of activation was also observed for TNFα in combination with either YB-1 or PGRN. However, stimulation with all three ligands combined (mTNFα, YB-1, and PGRN) inhibited the activation of ERK, p38, and NF-κB p65 in comparison to the TNFα-induced signal (Figure 3A).

To further validate our results, we utilized a commercial NF-κB reporter assay. Stimulation of the transfected human embryonic kidney cells with recombinant TNFα showed a robust induction of luciferase activity (Figure 3B). Similarly, stimulation with either YB-1 or PGRN alone also induced NF-κB luciferase activity, however, to a lesser extent of approximately 2-fold (Figure 3B). In agreement with the above results, adding either PGRN or YB-1 inhibited the TNF-induced reporter activity by 30% and 50% respectively, while combined stimulation with YB-1 and PGRN shows maximal inhibition (≈65%).

### 2.4. YB-1/PGRN Alters the TNFα-Induced Gene Expression Profile

**Figure 4:**
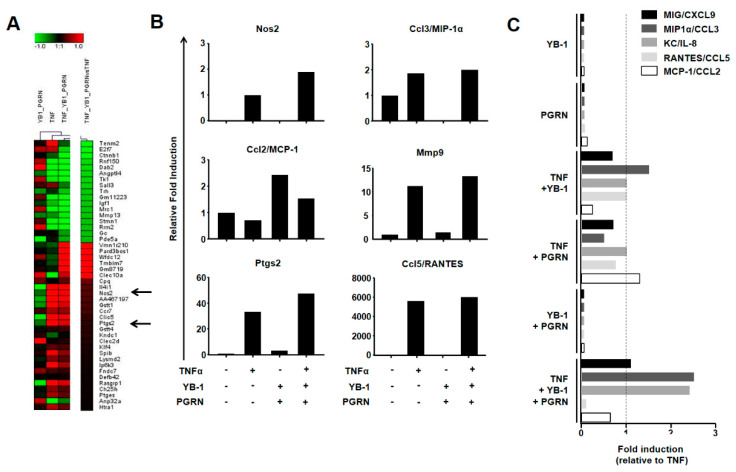
YB-1 and PGRN modulate TNF-induced cell responses. (**A**) BMDMs were stimulated as described above, lysed, and RNA extracted. Gene array analysis of the RNA was used to identify alterations in gene expression between stimulation conditions. Heat map visualization illustrates differentially expressed genes between each of the stimulation conditions compared to the negative control (i.e., unstimulated cells). Additionally, the comparison of TNFα alone with cells stimulated with TNFα, YB-1, and PGRN. Genes regulated >1.5 fold were selected. Nos2 and Ptgs2 are indicated (arrows). (**B**) BMDMs were stimulated with recombinant mTNF (625 pM), PGRN (5 nM), and YB-1 (28 nM) for 24 h. Cells were lysed; RNA was purified and transcribed into cDNA. Taqman gene expression analysis for NosS2, Ptgs2, MCP-1/CCL2, MIP1α/CCL3, RANTES/CCL5, and matrix metallopeptidase 9 (MMP9) were used to validate the results. Gene expression levels are illustrated as relative fold induction, using the gene expression of the negative control as 1. (**C**) BMDMs were stimulated with recombinant mTNF (625 pM), PGRN (5 nM), and YB-1 (28 nM) for 24 h. Cytokines secreted into the media were measured with a cytometric bead array and analyzed by FACS. The cytokines MIG/CXCL9, MIP1α/CCL3, MCP-1/CCL2, RANTES/CCL5, and KC/IL-8 were found to be regulated depending on the stimulus condition. Values are expressed as relative fold induction, using levels of TNFα-induced cytokine secretion as 1.

To gain a comprehensive overview of the changes going on within the cells, we extracted RNA and analyzed the gene expression profiles. The comparison of TNF-stimulated macrophages with unstimulated cells identified more than 1124 differentially expressed genes (cut off >1.5-fold), including 26 known TNF targets. Comparison of the triple stimulation with TNF alone identified a unique subset of 46 differentially expressed genes (Figure 4A). To validate these findings, we again purified RNA from stimulated cells, transcribed it into cDNA, and performed TaqMan expression analysis for six candidate genes (Nos2, Ptgs2, Ccl2/MCP-1, Ccl3/MIP1α, Ccl5/RANTES, and Mmp9). As seen in Figure 4B, we confirmed the results of two candidates selected from the gene array, namely Nos2 and Ptgs2, which both showed a further upregulation upon triple stimulation in comparison to TNF alone. A similar trend was seen for matrix metallopeptidase 9 (Mmp9), which is a known TNFR as well as YB-1 target gene [41,42]. In the multiplex assay, we observed an induction for both Ccl3/MIP1α and Ccl5/RANTES. The effect of YB-1/PGRN co-stimulation was less prominent. This is most likely because RNA is subject to additional regulation within the cell at the level of translation. Here, YB-1 is known to exert much of its activity [43]. YB-1 is able to both enhance and suppress the translation of chemokine mRNAs, such as Ccl5/RANTES, depending upon the cellular circumstances [44,45,46,47,48].

### 2.5. YB-1/PGRN-Mediated Inhibition Modulates TNF-Induced Cytokine/Chemokine Expression

Having demonstrated that YB-1/PGRN modulates TNFα-induced signaling, we next asked how this translates into a functional outcome for the cell. Since TNFα is regarded as the master regulator of immune-mediated inflammation, we first analyzed the cytokine/chemokine secretion of our wild-type BMDMs using a multiplex assay. As expected, 24 h after TNFα stimulation, we detected measurable levels of the pro-inflammatory chemokines CXCL9/MIG, CCL3/MIP1a, CXCL1/KC, CCL5/RANTES, and CCL2/MCP-1 in the cell supernatants (Figure 4C). Incubation with either YB-1 or PGRN alone showed no induction. Co-stimulation of TNFα with either YB-1 or PGRN showed subtle changes in chemokine secretion; however, the combined effect of three stimuli showed clear modulatory effects. The levels of MIP1α and KC were more than 2-fold enhanced, whereas CCL5/RANTES is ablated and MCP-1 is suppressed.

## 3. Discussion

Chemokine secretion is essential for the recruitment of immune cells, including monocytes/macrophages. These cells are not only important for the induction of inflammation, but they also contribute to wound healing and the restoration of tissue homeostasis. Indeed, we and others have recently shown that YB-1 plays an essential role in each of these stages of disease [9,46,49,50,51,52,53].

The current challenge lies in defining both the cell intrinsic activities of YB-1 as well as its extrinsic activities. YB-1 secretion is known to be induced by a number of pro-inflammatory stimuli, such as PDGF, TGF-β, as well as LPS, and extracellular YB-1 possesses chemoattractant activity [6,7,8,54]. Indeed, its ability to bind to cell surface receptors, such as receptor Notch-3, have led us to propose the existence of an auto-regulatory loop [55].

Herein, we have identified a new activity for extracellular YB-1, namely modulating TNFR activity. TNF is considered a master regulator of inflammation, due to its abilities to induce the production of a number of inflammatory mediators as well as proteases, which contribute to tissue damage as well as cancerogenesis [56,57].

Similar to YB-1, TNF also possesses the ability to auto-regulate its own activity. TNFR stimulation activates NF-κB, amongst other factors, which induces the expression of TNF mRNA. Recently, we identified YB-1 as an essential component of the TNFR signaling cascade leading to NF-κB activation, as TNF stimulation of YB-1-deficient cells failed to activate NF-κB, as was previously shown for both IGF-1 and IL-1β signaling [58,59,60]. A failure to activate YB-1 and thus NF-κB, negatively impacts on cell survival in monocytes, macrophages, and T cells [60,61,62].

However, this is not the only level where YB-1 may affect TNF functionality. The TNF mRNA possesses the same regulatory sequences (class II AU-rich elements) that are found in the mRNA of GM-CSF, which is a known target of YB-1 [63,64,65,66]. Additionally, RNA binding proteins, such as TIA-1, TIAR, and HuR, which are components of the stress granules, regulate TNF mRNA. Since YB-1 regulates stress granule formation and is found together with TIA-1, TIAR, and HuR within these structures, there is a strong possibility that YB-1 contributes to the regulation of TNF mRNA [67,68,69].

In addition to enhancing the expression of TNF mRNA, NF-κB also induces TRAF2 expression [32]. TRAF2 is a common signaling component of both TNFR1 and TNFR2 that plays an important role in regulating canonical *versus* non-canonical NF-κB activation, as well as the balance between survival and death signaling [70]. Thus, an induction of TRAF2 would amplify the canonical NF-κB pathway (p50/p65). Since TRAF2 is essential for activating p65, YB-1 must lay in between. The phosphorylation of TRAF2 by protein kinase C regulates its ability to recruit and activate IKKα, which in turn phosphorylates IκBα, targeting it for degradation and thereby activating NF-κB (p50/p65) [70,71]. In addition, it has recently been reported that TRAF2 mediates the recruitment of ubiquitin ligases to the TNFR1 complex I thereby promoting NF-κB activation [72]. Moreover, YB-1 has been shown to directly interact with p65 (RelA) and act as a transcriptional co-activator [73]. A number of kinases (RSK, AKT, ERK, PKC, CKII) have been identified that phosphorylate YB-1 within its cold shock domain, thereby inducing nuclear translocation [74,75]. Thus, it is foreseeable that the TNF receptor oligomerization induced by ligand binding results in the recruitment and oligomerization of adaptors, such as TRAF2, which in turn recruit kinases and ubiquitin ligases that activate both NF-κB and its *trans*-activator YB-1.

In this study, we focused on stimulation of the TNFRs with soluble TNF, which activates TNFR1 [76]. Recently, it was shown that TNFR1 oligomerization is essential to induce signaling and that this requires both dimerization of receptor chains via the preligand assembly domain and ligand binding [77]. Therefore, it is conceivable that YB-1/PGRN interferes with oligomer formation and thereby prevents signal transduction. Determining the structure of these interactions is essential, as this may reveal new targets for development as anti-TNF therapy, as current therapies although successful have their limitations [78]. How YB-1/PGRN influence membrane-bound ligand activity requires further study.

Recently, PGRN was reported to activate Notch signaling pathways in neurons [79]. YB-1 shows abundant expression in neurons and is a ligand for receptor Notch-3 [7,80]. Since TNF and TNFR interactions are proposed to play a role in neuronal growth, the disruptions of these interactions may contribute to cognitive impairment, as seen in patients with progranulin mutations [81,82]. Finally, progranulin was recently identified as a ligand for ephrin receptor A2; however, no interaction with TNFR1 was found, suggesting that these receptors function independently, at least with regard to ligand binding [83].

## 4. Materials and Methods

### 4.1. Recombinant Proteins

Recombinant human PGRN (AG-40A-0068 from Adipogen, San Diego, CA, USA) was used for cell stimulation experiments with TNFα, YB-1, and PGRN. Recombinant murine TNFα (mTNFα) was purchased from Peprotech (315-01A, Hamburg, Germany). Recombinant YB-1 was produced in our laboratory by the transient transfection of HEK293T cells, which were cultivated in DMEM growth media (+10% fetal calf serum (FCS), 1% penicillin/streptomycin). For transfection, 3 × 10^6^ cells were seeded in 10 cm petri dishes and grown to 70–80% confluence. Using the calcium phosphate method, cells were transfected with Flag-tagged human YB-1 (pcDNA3/Flag-YB-1), which was a gift of K. Kohno [84]. Media was changed after 5 h and cultivated for additional 48 h in 37 °C and 5% CO_2_. Cells were lysed for Flag-YB-1 purification with RIPA lysis buffer (50 mM Tris Base, 150 mM NaCl, 1 mM EDTA, 1% NP-40, 0.25% Sodium deoxycholate) supplemented with complete mini protease inhibitor cocktail (Roche) and Phos-Stop (Roche, Mannheim, Germany). Flag-YB-1 was purified with anti-DYKDDDDK G1 affinity resin (L00432, Genscript, Piscataway, NJ, USA) following the recommendation of the company. Flag-peptide (100 µg/mL) was used to elute Flag-YB-1 from the affinity resin. After dialysis in polyethylene glycol solution (average molecular weight 20 kDa), purified protein was shock frozen and stored at −80 °C until use (see Supplementary Figure S1). Purified protein was quantified using a Lowry assay (BioRad, Feldkirchen, Germany). Activity of rYB-1 was tested using a scratch assay [6].

GST-tagged YB-1 deletion mutants (a gift of K. Kohno) were used to identify the minimal sequence required for the interaction of YB-1 and TNFR-binding [84]. In contrast to GST-full length YB-1, GST-YB-1Δ1 is missing the N-terminal sequence of YB-1 (aa 1–50), the GST-YB-1Δ3 construct is missing the N-terminal sequence and the cold shock domain (aa 1–128), and the GST-YB-1Δ4 is missing the C-terminal sequence (aa 129–324). The GST-YB-1Δ5 only contains the cold shock domain (aa 51–128). A GST-tag construct was used as control. GST-tagged YB-1 deletion mutants were expressed in BL21 *E. coli* bacteria, bacteria were lysed, and the proteins purified with Glutathione Sepharose 4B (GE Healthcare, Chicago, IL, USA) and eluted with increasing glutathione concentrations, dialyzed, and analyzed by Western blot. Recombinant proteins were stored at −80 °C until use.

### 4.2. Cell Culture

#### 4.2.1. Bone Marrow-Derived Macrophages (BMDMs)

BMDMs were generated from wild-type mice by flushing femur and tibia with sterile DPBS. Erythrocytes were lysed under hypotonic conditions and cells seeded with 2 × 10^6^ cells/mL in RPMI growth media supplemented with 10% FCS, 1% penicillin/streptomycin, and 10 ng/mL murine macrophage colony-stimulating factor (M-CSF, 315-02, Peprotech). Cells were cultivated under humidified conditions at 37 °C and 5% CO_2_. Cells were fed every 2 days until full differentiation after 7 days. Then, cells were starved overnight and stimulated with either 28 nM YB-1, 5 nM PGRN, or 625 pM TNFα alone or in combination for the time period indicated. Following stimulation, the cells were lysed in DISC lysis buffer (30 mM Tris pH 7.4, 120 mM NaCl, 10% Glycerin, 1% Triton-X100) containing complete mini protease inhibitor cocktail and Phos-Stop. Then, lysates were separated by SDS-PAGE and analyzed by Western blotting.

Mice were housed according to FELASA guidelines (*Federation of European Laboratory Animal Science Association*) in a 12 h/12 h light dark cycle at 22 °C in the Central Animal Facility of the Otto-von-Guericke University Magdeburg under specific pathogen-free (SPF) conditions using individual ventilated cages (IVC, Techniplast, Buguggiate, Italy) with food and water *ad libitum*. Experiments were conducted according to the German National Guidelines for the Use of Experimental Animals (Animal Protection Act) and approved by the State of Saxony-Anhalt (AZ UniMD 42502-2-1401, 4 October 2016).

#### 4.2.2. Cell Lines

All cell lines were obtained from the ATCC. RAW264.7 is a monocytic macrophage cell line derived from ascites of a tumor induced by the intraperitoneal injection of Abelson Leukaemia Virus. Cells were cultivated in DMEM growth media (+10% FCS, 1% penicillin/streptomycin) under humidified conditions at 37 °C and 5% CO_2_. THP-1 is a human monocytic cell line derived from an acute monocytic leukemia patient. Cells were cultivated in RPMI growth media supplemented with 10% FCS, 1% penicillin/streptomycin, and 50 µM β-mercaptoethanol under humidified conditions at 37 °C and 5% CO_2_. Rat mesangial cells were cultivated in RPMI growth media containing 10% FCS, 1% penicillin/streptomycin under humidified conditions at 37 °C, and 5% CO_2_. HEK293T, a human embryonic kidney cell line, was cultivated in DMEM growth media containing 10% FCS and 1% penicillin/streptomycin under humidified conditions at 37 °C and 5% CO_2_.

### 4.3. Competitive TNFα-Binding Assay

RAW264.7 macrophages were used for competitive TNFα-binding assay analyzed by flow cytometry. Cells were detached, washed with DPBS, and pre-incubated for 30 min at 4 °C with increasing doses of recombinant Flag-YB-1 (0.14, 0.7, 1.4, 3.5 µM), GST-YB-1 deletion constructs (2 µM) to map the region of interaction, and combined Flag-YB-1 and PGRN. After pre-incubation, cells were stained with 0.016 µM biotinylated TNFα (Bt-TNFα) for 30 min, followed by Avidin-FITC for 30 min at 4 °C according to the manufacturer’s instructions (Fluorokine Biotinylated Human TNFα Kit, NFTA0, R&D systems, Minneapolis, MN, USA) and analyzed by flow cytometry. THP-1 cells and rat mesangial cells were cultivated with RPMI growth media (+10% FCS, 1% penicillin/streptomycin) and pre-incubated for 30 min at 4 °C with 2 µM Flag-YB 1. Afterwards, they were stained with Bt-TNFα and FITC-Avidin as described above and analyzed by flow cytometry.

### 4.4. Western Blot Analysis

Western blots were performed as previously published [45]. Primary antibodies were incubated over night at 4 °C and diluted according to the manufacturer’s instructions. The following antibodies were used: anti-pERK 1:1000 rabbit (4370, Cell Signaling, Danvers, MA, USA), anti-pp38 1:1000 rabbit (9211, Cell Signaling), anti-NF-κB 1:1000 mouse (6956, Cell Signaling), anti-pNF-κB 1:1000 rabbit (3033, Cell Signaling), anti-YB-1 1:1000 rabbit (Eurogentec, Seraing, Belgium), biotinylated anti-YB-1 mouse 1:1000 (self-made), anti-actin 1:2000 mouse (A5441, Sigma, Darmstadt, Germany), anti-GAPDH (2118, Cell Signaling), and anti-GST 1:1000 rabbit (2622, Cell Signaling). Membranes were washed with TBST and probed with the corresponding horseradish peroxidase-conjugated secondary antibodies for 2 h at room temperature (goat-anti-mouse IgG (H+L)-HRP (1031-05) or goat-anti-rabbit IgG (H+L)-HRP (4050-05) 1:5000, Southern Biotech). After additional washing, the membranes were visualized using Pierce ECL Western Blotting Substrate (Thermo Fisher Scientific, Rockford, IL, USA). Images were captured using an Intas ChemoCam Imager System and analyzed and quantified with the LabImage 1D Software (Intas, Göttingen, Germany). Values are presented as relative band intensities (RBI) with the value of TNF stimulation set to 100%.

### 4.5. Quantification of Serum YB-1 Levels

The level of YB-1 expression in patient samples was quantified by Western blotting using purified recombinant human YB-1 to generate a standard curve (see Supplementary Figure S2). Controls have serum levels of 140 to 280 µM; levels are comparable to the α-, β-, and γ-globulin fractions. In patient serum, the level is increased 3 to 6-fold.

### 4.6. NF-κB Luciferase Activity

The luciferase reporter assay was performed using the pGL4.32 vector (Promega, Madison, WI, USA) and BrightGlo Luciferase Assay System (Promega) according to the manufacturer’s instructions.

### 4.7. Yeast Two-Hybrid Screen

A yeast two-hybrid screen (Proquest, Invitrogen, Carlsbad, CA, USA) was performed as previously described [37]. 2 × 10^6^ clones were analyzed to identify YB-1 interacting proteins. To identify positive clones, four selection criteria were used: growth on histidine- and uracil-deficient media, growth inhibition on media containing 5-FOA, and enzymatic reaction with X-GAL. Positive clones were selected, sequenced, and analyzed using [85].

### 4.8. RNA Analysis

BMDMs were prepared as described above and stimulated with TNF, YB-1, and/or PGRN in various combinations for 24 h. Cells were lysed and RNA was isolated with an RNeasy mini kit (QIAGEN, Hilden, Germany) following the instructions of the supplier. For gene expression screening, RNA was analyzed by microarray analysis as described previously [45]. Shortly, Cyanine-3 (Cy3) labeled cRNA was prepared using the One-Color Low RNA Input Linear Amplification PLUS kit (Agilent) according to the manufacturer’s instructions, followed by RNAeasy column purification (QIAGEN). Cy3-labeled cRNA was fragmented at 60 °C for 30 min and hybridized to Agilent Whole Mouse Genome Microarrays 4 x44k V2 026655 for 17 h at 65 °C in a rotating Agilent hybridization oven. After hybridization, microarrays were washed and dried immediately by brief centrifugation. Scanned images were analyzed with Feature Extraction Software 10.5 (Agilent, Santa Clara, CA, USA) using default parameters to obtain background subtracted and spatially detrended processed signal intensities. For analysis, log2-transformed expression values were used to select genes regulated +/−1.5 fold compared to the negative control. Selected genes were secondly checked for differential expression between TNF alone versus TNF, YB-1, and PGRN-stimulated BMDMs. Selected genes were clustered hierarchically using Genesis 1.7.7 software (Graz University of Technology).

To validate gene expression data from microarray analysis, Taqman quantitative PCR analysis was performed as previously described [45]. For this, cDNA was generated using a Revert Aid First Strand cDNA Synthesis Kit (K1622, Thermo Fisher Scientific) following the instructions of the manufacturer. The following Taqman gene expression assays from Thermo Fisher Scientific were used: Tmbim7 (Mm00512517_m1), Nos2 (Mm00440502_m1), Ptgs2 (Mm00478374_m1), Ccl2 (Mm00441242_m1), Ccl3 (Mm00441259_g1), Ccl5 (Mm01302427_m1), Actin (Mm00607939_s1), 18S (Mm03928990_g1), Mmp9 (MM00442991_m1).

### 4.9. Cytokine Measurement

Cytokine expression into the media secreted by stimulated BMDMs was measured with a BD cytometric bead array (for the cytokines/chemokines CCL5/RANTES, KC, MIG, CCL3/MIP 1α, CCL2/MCP-1, TNFα, and IL 6) as previously described [45]. Cells were stimulated as described above and media were collected, centrifuged, immediately frozen with liquid nitrogen, and stored at -80 °C. Samples were mixed with capture beads according to the instructions of the supplier, incubated for 1 h in the dark, and washed several times before analysis with flow cytometry.

### 4.10. Statistical Analysis

All results were obtained and confirmed in at least two independent experiments performed in triplicate, if not otherwise stated. Results were calculated and presented as means ± SD. The Student t-test was applied for two-group comparisons * *p* = 0.05, ** *p* = 0.01, *** *p* = 0.001 were considered as statistically significant (n.s. was considered as non-significant).

## Supplementary Materials

Supplementary Materials can be found at https://www.mdpi.com/1422-0067/21/19/7076/s1. Figure S1: Purification of recombinant YB-1; Figure S2: Quantification of serum YB-1.

## Abbreviations

BMDMs	bone marrow-derived macrophages
CCL2/MCP-1	chemokine (C-C motif) ligand 2/monocyte chemotactic protein 1
CCL3/MIP1α	chemokine (C-C motif) ligand 3/macrophage inflammatory protein 1-alpha
CCL5/RANTES	chemokine (C-C motif) ligand 5
CSD	cold shock domain
CXCL1/KC	chemokine (C-X-C motif) ligand 1
CXCL9/MIG	chemokine (C-X-C motif) ligand 9/monokine induced by gamma interferon
ERK	extracellular-signal regulated kinase
FCS	fetal calf serum
GST	glutathione S-transferase
MMP-9	matrix metalloproteinase-9
NF-κB	nuclear factor kappa-B
NOS2	nitric oxide synthase 2
PGRN	progranulin
PTGS2/COX2	prostaglandin-endoperoxidase synthase 2/cyclooxygenase 2
TNFα	tumor necrosis factor alpha
TNFR1/2	tumor necrosis factor receptor 1/2
YB-1	Y-box binding protein-1
