# Supplementary material for: YB-1 Interferes with TNFα–TNFR Binding and Modulates Progranulin-Mediated Inhibition of TNFα Signaling
Source: Int J Mol Sci. 2020 Sep 25;21(19):7076. doi: 10.3390/ijms21197076 (PMC7583764; doi:10.3390/ijms21197076)
Supplement: Supplementary file 1 [file ijms-21-07076-s001.pdf]

International Journal of *Molecular Sciences*

MDPI

Supplementary

YB-1 Interferes with TNFα–TNFR Binding and Modulates Progranulin-Mediated Inhibition of TNFα Signaling

Christopher L. Hessman [1,†], Josephine Hildebrandt [1,†], Aneri Shah [1], Sabine Brandt [1], Antonia Bock [1], Björn C. Frye [2,‡], Ute Raffetseder [2], Robert Geffers [3], Monika C. Brunner-Weinzierl [4], Berend Isermann [5], Peter R. Mertens [1,*] and Jonathan A. Lindquist [1,*]

1 Clinic of Nephrology and Hypertension, Diabetes and Endocrinology, Otto-von-Guericke University, 39120 Magdeburg, Germany; c.hessman@gmx.net (C.L.H.); josephine.hildebrandt@freenet.de (J.H.); aneri.shah@ovgu.de (A.S.); Sabine.Brandt@med.ovgu.de (S.B.); antonia.bock94@t-online.de (A.B.)
2 Department of Nephrology and Clinical Immunology, RWTH Aachen University, 52074 Aachen, Germany; uraffetseder@ukaachen.de (B.C.F.); uraffetseder@ukaachen.de (U.R.)
3 Genome Analytics Research Group, Helmholtz Centre for Infection Research, 38124 Braunschweig, Germany; Robert.Geffers@helmholtz-hzi.de
4 Department of Experimental Pediatrics, Otto-von-Guericke University, 39120 Magdeburg, Germany; Monika.Brunner-Weinzierl@med.ovgu.de
5 Institute of Laboratory Medicine, Clinical Chemistry and Molecular Diagnostics, University Hospital Leipzig, 04103 Leipzig, Germany; Berend.Isermann@medizin.uni-leipzig.de
‡ Present address: Department of Pneumology, Medical Center - University Hospital Freiburg, 79106 Freiburg, Germany; bjoern.christian.frye@uniklinik-freiburg.de
† These authors contributed equally to this work.
* Correspondence: Peter.Mertens@med.ovgu.de (P.R.M.); Jon.Lindquist@med.ovgu.de (J.A.L.); Tel.: +49-391-6713236 (P.R.M.); +49-391-6724703 (J.A.L.)

Received: 4 September 2020; Accepted: 22 September 2020; Published: date

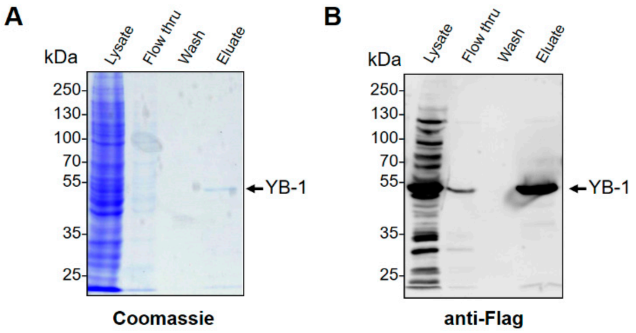

Supplementary Figure 1. Purification of recombinant YB-1. (A) HEK293T cells were transformed with a plasmid encoding Flag-tagged human YB-1 [84]. A Coomassie stained gel shows the various steps of the purification procedure (see Methods). (B) The integrity of recombinant human YB-1 (50 kDa) was determined by Western blotting.

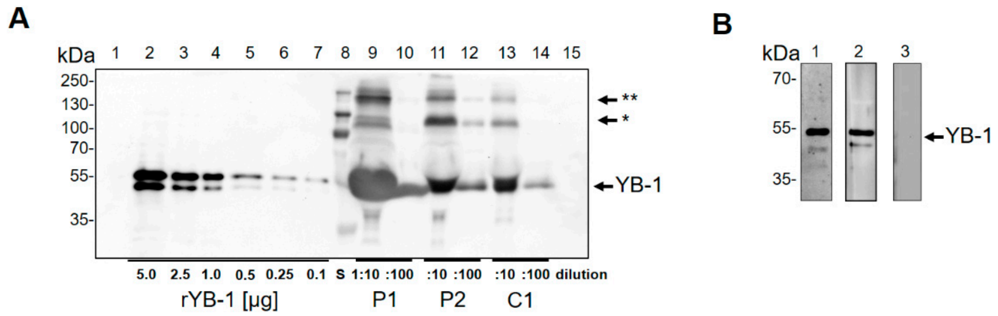

Supplementary Figure 2. Quantification of serum YB-1. Recombinant human YB-1 was loaded to generate a standard curve (lanes 2–7) for quantification. Flag-tagged YB-1 runs at a slightly higher MW than the endogenous YB-1 protein. Serum from two patients (P1, P2) and one healthy control (C1) were diluted as indicated. The presence of YB-1 in serum was detected by Western blotting using anti-YB-1. (B) Antibody specificity is demonstrated by western blotting: lane 1 – anti-Flag, lane 2 – anti-YB-1, and lane 3 – secondary antibody alone.

© 2020 by the authors. Submitted for possible open access publication under the terms and conditions of the Creative Commons Attribution (CC BY) license (http://creativecommons.org/licenses/by/4.0/).
